# Supplementary material for: Structural insights into respiratory complex I deficiency and assembly from the mitochondrial disease-related ndufs4−/− mouse
Source: EMBO J. 2024 Jan 2;43(2):4. doi: 10.1038/s44318-023-00001-4 (PMC10897435; doi:10.1038/s44318-023-00001-4)
Supplement: Supplementary file 1 — Appendix [file 44318_2023_1_MOESM1_ESM.pdf]

Appendix Data for:

Structural insights into complex I deficiency and assembly from the disease-related *ndufs4*$^{-/-}$ mouse

Zhan Yin†, Ahmed-Noor A. Agip‡, Hannah R. Bridges*, and Judy Hirst*

The Medical Research Council Mitochondrial Biology Unit, University of Cambridge, Keith Peters Building, Cambridge Biomedical Campus, Cambridge, United Kingdom

† Current address: Department of Biochemistry, University of Cambridge, Tennis Court Road, Cambridge, CB2 1GA, UK

‡ Current Address: Max-Planck-Institute of Biophysics, Frankfurt 60438, Germany

* e-mail: hrb@mrc-mbu.cam.ac.uk; jh480@cam.ac.uk

Contents

Appendix Figure S1: Cryo-EM data collection and structural reconstruction of complex I from *ndufs4*$^{-/-}$ mouse kidney…………………………………………………………………………………………………....3

Appendix Figure S2: Cryo-EM data collection and structural reconstruction of complex I from *ndufs4*$^{-/-}$ mouse heart……………………………………………………………………………………………………...4

Appendix Figure S3: Subclassification of polished particles in cryoSPARC by 3D classification………………..5

Appendix Figure S4: Local resolution maps, FSC curves and angular distribution plots for structural data on *ndufs4*$^{-/-}$ heart complex I…………………………………………………………………………………....6

Appendix Figure S5: Ubiquinone binding in the *ndufs4*$^{-/-}$ heart complex I………………………………………..7

Appendix Table S1: Peptide-based protein identification of the composition of *ndufs4*$^{-/-}$ complex I purified from heart……………………………………………………………………………………………….....…9

Appendix Table S2: Peptide-based protein identification of the composition of *wild-type* complex I purified from heart…………………………………………………………………………………………….............10

Appendix Table S3: Cryo-EM data collection, refinement and validation statistics for maps and models of *ndufs4*$^{-/-}$ complex I and associated proteins……………………………………………………………......11

Appendix Table S4: Map-map correlations for consensus *ndufs4*$^{-/-}$ class 1-3 maps against reference *wild-type* active and deactive maps………………………………………………………………………………………12

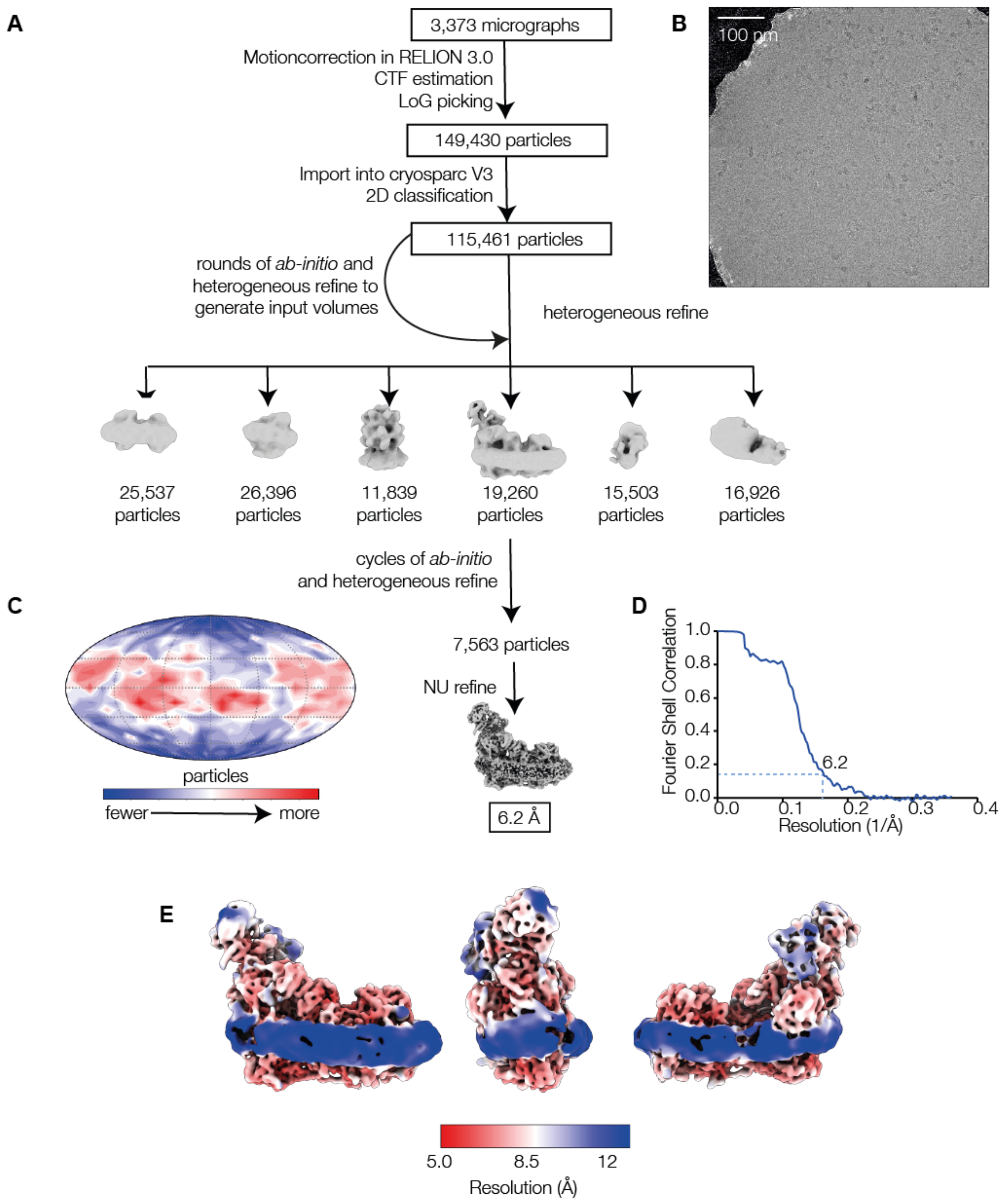

Figure S1. Cryo-EM data collection and structural reconstruction of complex I from *ndufs4*$^{-/-}$ mouse kidney. A) Data processing scheme for the mouse complex I from *ndufs4*$^{-/-}$ mouse kidney; B) example micrograph; C) angular distribution of particles; D) the global resolution estimate from the masked Fourier Shell Correlation curve is 6.2 Å at FSC = 0.143; and E) local resolution map calculated in cryoSPARC using an FSC = 0.5 cut-off.

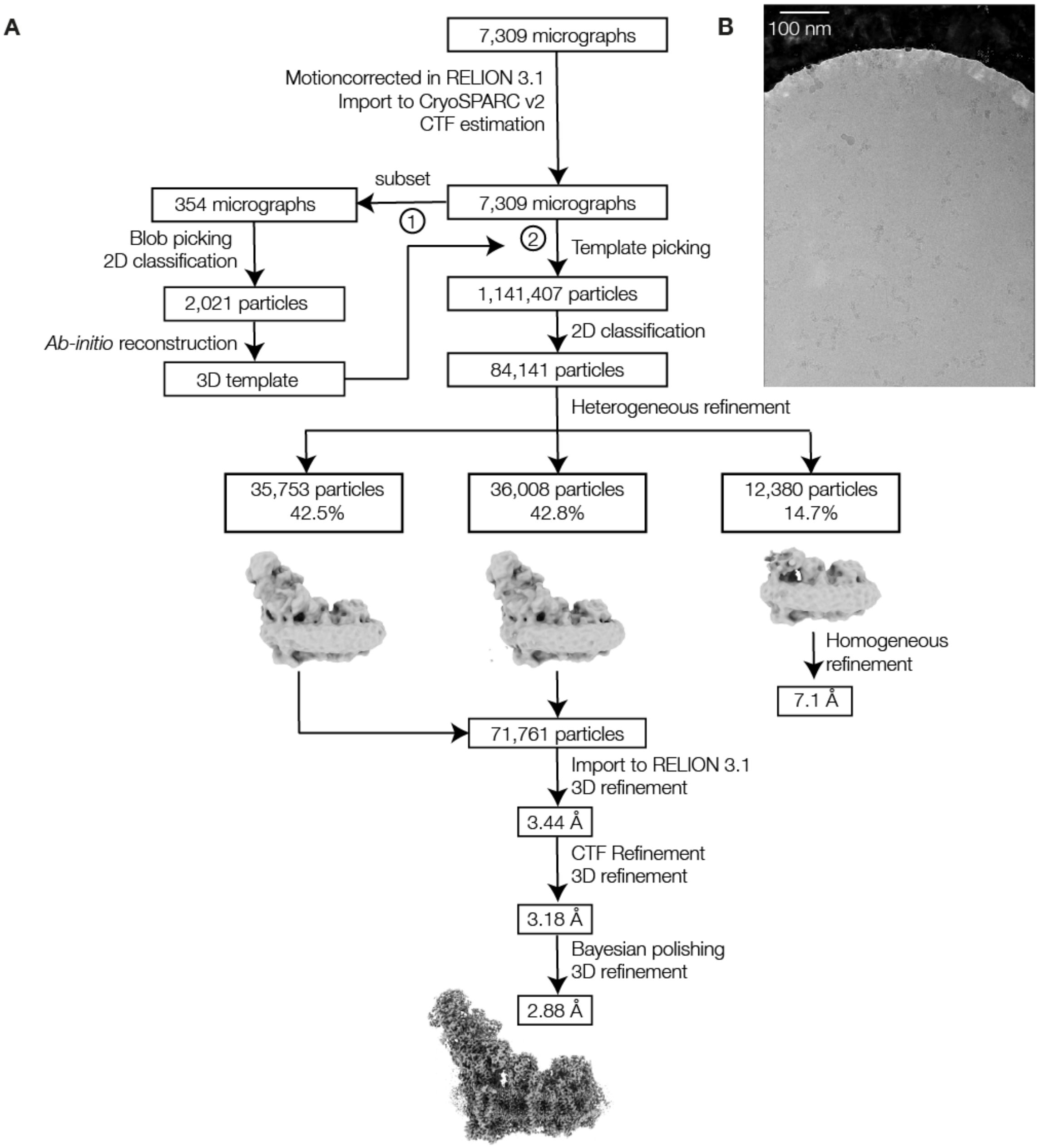

Figure S2. Cryo-EM data collection and structural reconstruction of complex I from *ndufs4*$^{-/-}$ mouse heart. A) Data processing scheme for the mouse complex I from *ndufs4*$^{-/-}$ mouse heart; B) example micrograph.

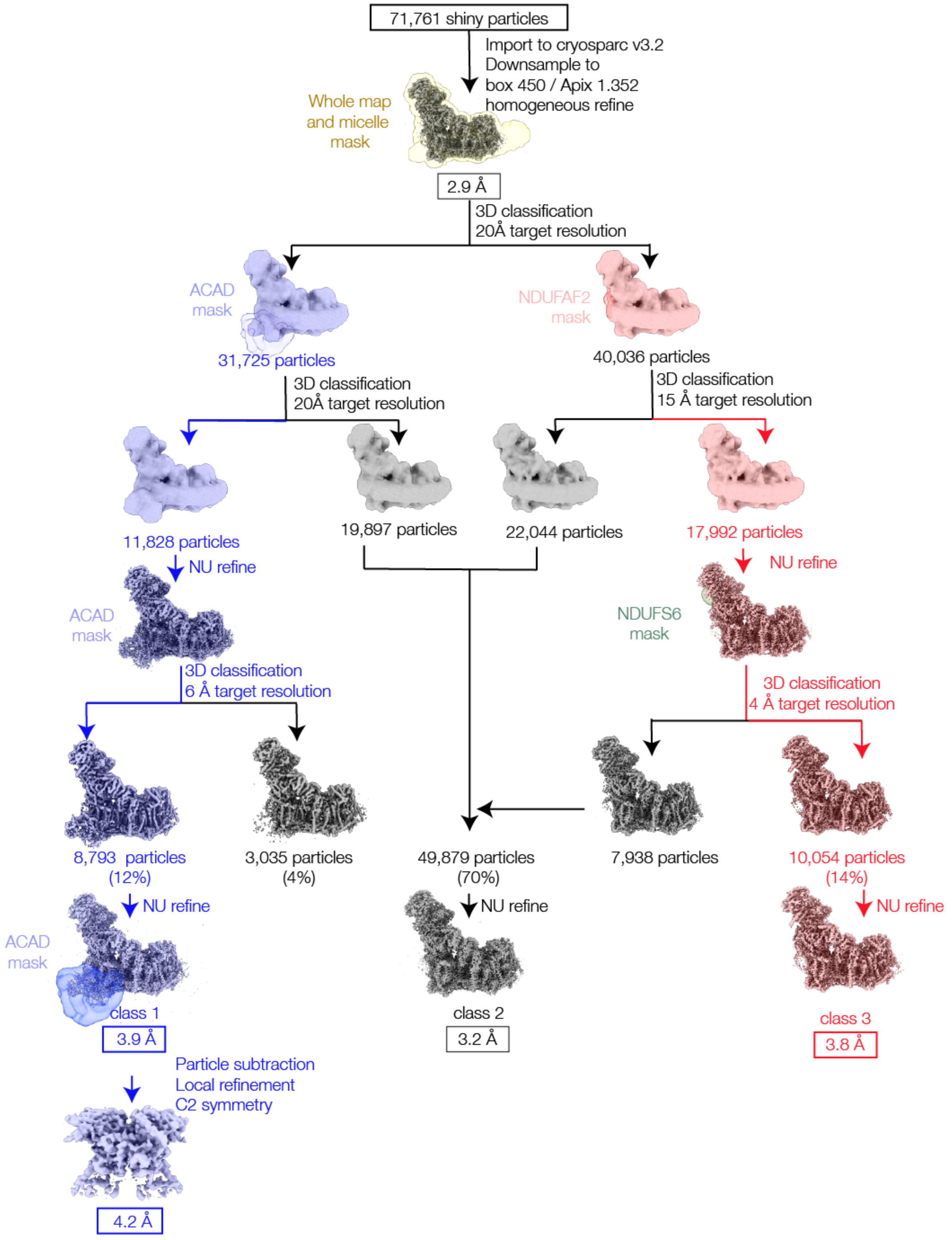

Figure S3. Subclassification of polished particles in cryoSPARC by 3D classification. After two rounds of classification (blue and pink) the intermediate (top) pink species had poor density for both NDUFS6 and NDUFAF2. Further subclassification around subunit NDUFS6 then showed that the molecules in the pink population contain either NDUFS6 (grey) or NDUFAF2 (pink), but not both.

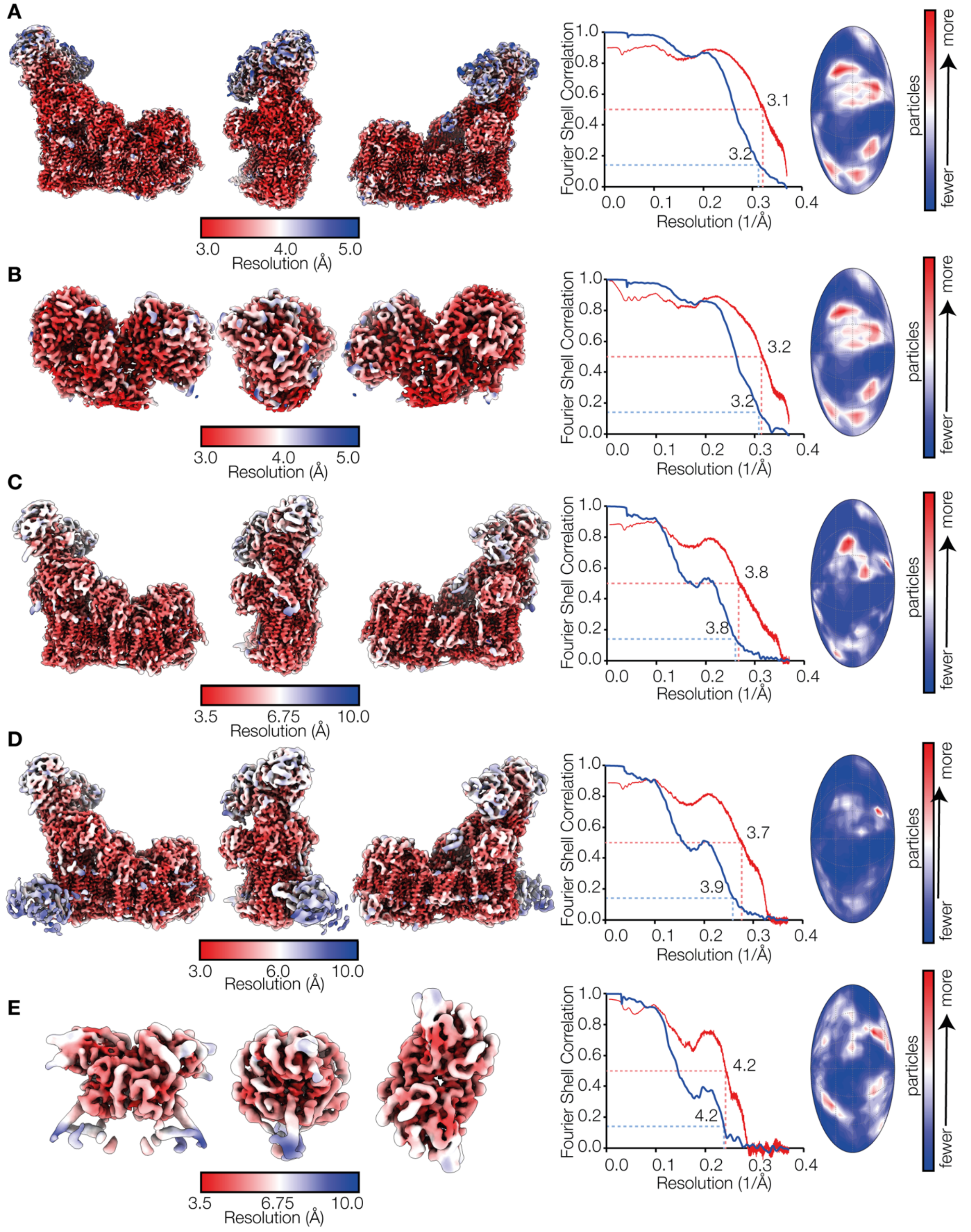

Figure S4. Local resolution maps, FSC curves and angular distribution plots for structural data on *ndufs4$^{-/-}$* heart complex I. Local resolutions were calculated in cryoSPARC using an FSC = 0.5 cut-off. Half-map (blue) and map-model (red) FSC curves are shown with the resolution at FSC = 0.143 indicated for the half map FSC, and FSC = 0.5 is indicated for the map-model FSC. Data are shown for A) class 2 *ndufs4$^{-/-}$* complex I; B) class 2 *ndufs4$^{-/-}$* N-module of complex I; C) class 3 *ndufs4$^{-/-}$* complex I; D) class1 *ndufs4$^{-/-}$* complex I; E) class 1 *ndufs4$^{-/-}$* ACADVL only.

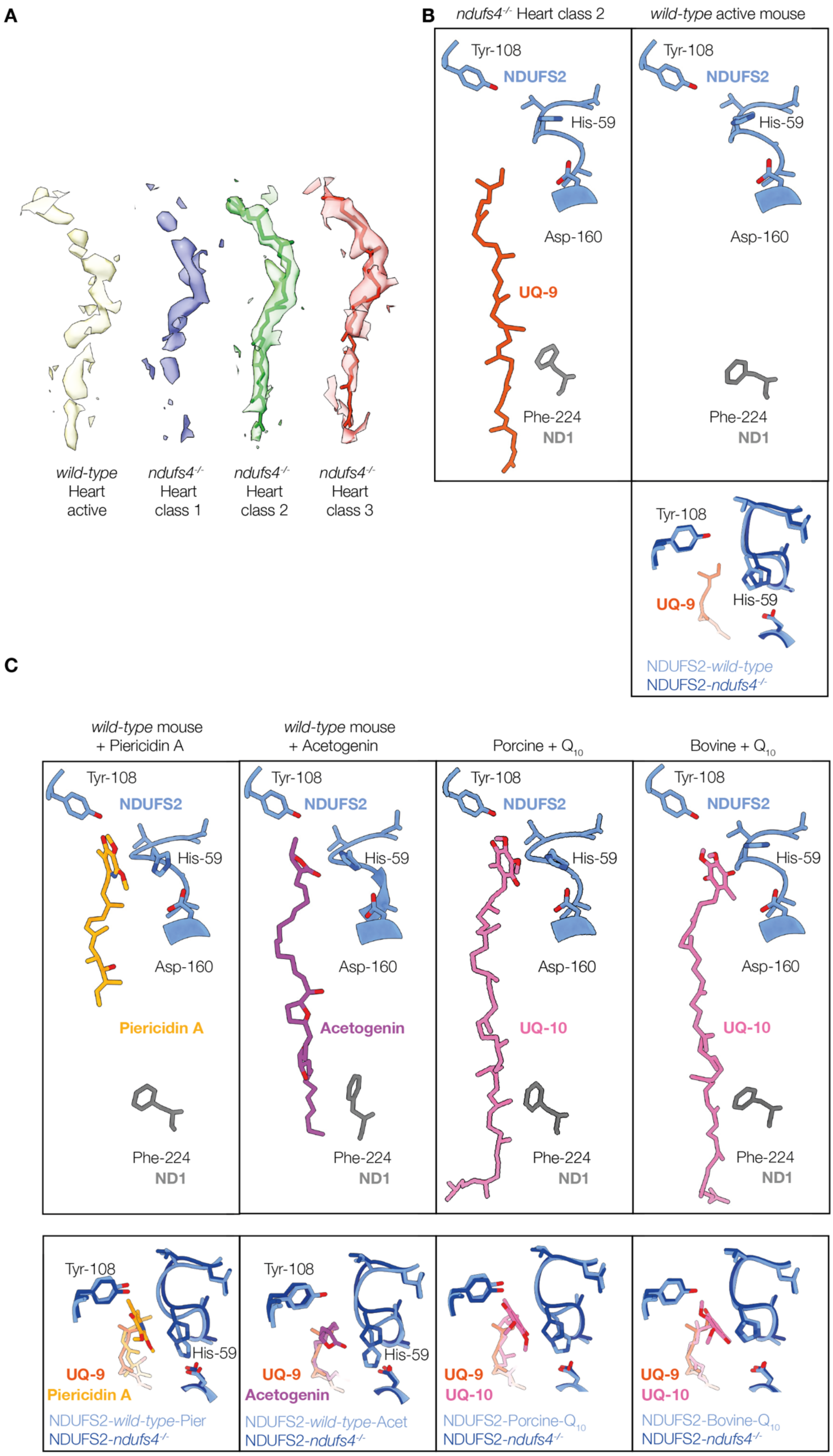
A
wild-type Heart active
ndufs4-/- Heart class 1
ndufs4-/- Heart class 2
ndufs4-/- Heart class 3
B
ndufs4-/- Heart class 2
wild-type active mouse
Tyr-108
NDUFS2
His-59
Asp-160
UQ-9
Phe-224
ND1
NDUFS2-wild-type
NDUFS2-ndufs4-/-
C
wild-type mouse + Piericidin A
wild-type mouse + Acetogenin
Porcine + Q_{10}
Bovine + Q_{10}
Piericidin A
Acetogenin
UQ-10
NDUFS2-wild-type-Pier
NDUFS2-wild-type-Acet
NDUFS2-Porcine-Q_{10}
NDUFS2-Bovine-Q_{10}
NDUFS2-ndufs4-/-

Figure S5. Ubiquinone binding in the *ndufs4$^{-/-}$* heart complex I. A) Cryo-EM density in the ubiquinone-binding channel in the density maps for *wild-type* active mouse complex I (EMD: 11377 (Bridges *et al*, 2020)) and *ndufs4$^{-/-}$* heart complex I classes 1 to 3 (EMD: 16398, 16516, 16518). B) Key ubiquinone-binding site elements in *ndufs4$^{-/-}$* heart class 2 (PDB: 8CA3) and the active *wild-type* reference structure (PDB: 6ZR2) (Bridges *et al*, 2020) and a comparison of structures in the headgroup-binding region. C) Key ubiquinone-binding site elements in structures of *wild-type* mouse with piericidin (PDB: 6ZTQ) (Bridges *et al*, 2020), *wild-type* mouse with acetogenin (PDB: 7PSA) (Grba *et al*, 2022), bovine complex I with ubiquinone-10 (7QSL) (Chung *et al*, 2022b), and porcine complex I with ubiquinone-10 (PDB: 7V2C) (Gu *et al*, 2022), and comparisons of structures with *ndufs4$^{-/-}$* heart class 2 in the headgroup-binding region.

Subunit	MALDI TOF/TOF MS/MS			LC MS		
	Unique peptides	Sequence coverage	Score	Unique peptides	Sequence coverage	Score/32
NDUFA1	4	42%	76/34	5	42%	109
NDUFA2	4	32%	238/33	9	70%	161
NDUFA3	3	32%	40/34	4	50%	74
NDUFA5	3	31%	109/35	10	63%	318
NDUFA6	3	26%	148/33	19	87%	546
NDUFA7	-	-	-	18	85%	613
NDUFA8	4	26%	127/34	11	48%	333
NDUFA9	11	35%	596/35	25	64%	1050
NDUFA10	11	42%	627/34	20	41%	699
NDUFA11	-	-	-	2	10%	146
NDUFA12	-	-	-	13	77%	460
NDUFA13	5	30%	230/35	20	81%	1165
NDUFAB1	-	-	-	1	11%	55
NDUFB1	3	45%	116/34	5	50%	56
NDUFB2	2	8%	70/33	1	8%	41
NDUFB3	2	14%	39/35	5	33%	88
NDUFB4	7	53%	302/33	17	69%	488
NDUFB5	-	-	-	9	35%	316
NDUFB6	-	-	-	15	75%	260
NDUFB7	-	-	-	5	45%	405
NDUFB8	5	38%	273/34	9	43%	479
NDUFB9	3	24%	78/34	16	66%	381
NDUFB10	1	8%	54/34	11	55%	400
NDUFB11	1	7%	41/30	11	56%	472
NDUFC2	3	17%	126/33	12	60%	251
NDUFS1	6	13%	245/32	44	47%	2231
NDUFS2	12	30%	476/35	23	48%	809
NDUFS3	6	30%	336/34	18	46%	796
NDUFS5	-	-	-	10	46%	195
NDUFS6	4	39%	189/35	6	50%	466
NDUFS7	7	14%	219/34	10	30%	332
NDUFS8	-	-	-	8	30%	173
NDUFV1	11	27%	475/35	24	41%	731
NDUFV2	-	-	-	13	45%	804
NDUFV3	-	-	-	6	45%	121
ND1	-	-	-	3	8%	116
ND2	1	4%	61/33	-	-	-
ND4	4	7%	112/35	3	7%	73
ND5	1	2%	50/34	5	8%	217
ACAD9	2	4%	37/33	4	8%	147
ACADVL	10	21%	407/34	28	45%	1241
NDUFAF2	3	23%	149/35	11	71%	273

Table S1. Peptide-based protein identification of the composition of *ndufs4$^{-/-}$* complex I purified from heart. The number of peptides detected from each subunit is given together with the sequence coverage, relative to the sequence of the immature protein. The score given for each subunit is the sum of the peptide scores, with the denominator representing the 95% confidence threshold ($p < 0.05$). Subunits NDUFC1, ND3, ND4L and ND6 (as well as NUDFS4) were not detected.

Subunit	MALDI TOF/TOF MS/MS			LC MS		
	Unique peptides	Sequence coverage	Score	Unique peptides	Sequence coverage	Score/21
**NDUFA1**	2	14%	57/34	3	32%	63
**NDUFA2**	5	38%	191/33	10	72%	351
**NDUFA3**	2	21%	74/34	1	10%	26
**NDUFA5**	3	45%	209/34	3	29%	100
**NDUFA6**	4	32%	203/32	12	80%	520
**NDUFA7**	-	-	-	13	78%	725
**NDUFA8**	5	39%	275/33	3	16%	136
**NDUFA9**	13	37%	836/34	15	51%	608
**NDUFA10**	11	40%	723/33	6	21%	237
**NDUFA11**	-	-	-	-	-	-
**NDUFA12**	2	6%	59/33	12	64%	400
**NDUFA13**	3	22%	138/35	13	71%	649
**NDUFAB1**	4	21%	216/34	-	-	-
**NDUFB1**	2	31%	85/33	3	33%	58
**(NDUFB2)**	2	8%	52/33	1	8%	61
**(NDUFB3)**	3	14%	50/33	4	22%	74
**NDUFB4**	6	46%	298/33	9	61%	427
**NDUFB5**	3	19%	114/33	6	32%	175
**NDUFB6**	5	40%	175/33	9	57%	292
**NDUFB7**	2	10%	134/30	2	25%	227
**NDUFB8**	8	49%	449/33	5	37%	302
**NDUFB9**	-	-	-	9	50%	220
**NDUFB10**	4	21%	138/33	7	40%	271
**NDUFB11**	-	-	-	7	42%	165
**NDUFC2**	4	27%	166/34	7	45%	340
**NDUFS1**	12	20%	653/32	32	41%	1653
**NDUFS2**	10	31%	729/33	14	34%	519
**NDUFS3**	12	49%	818/33	10	33%	509
**NDUFS4**	7	30%	449/33	8	34%	284
**NDUFS5**	1	9%	85/34	5	43%	73
**NDUFS6**	4	32%	278/33	4	41%	258
**NDUFS7**	6	14%	217/33	5	24%	280
**NDUFS8**	5	24%	318/32	3	16%	136
**NDUFV1**	12	27%	574/33	18	32%	445
**NDUFV2**	7	25%	311/33	8	33%	526
**NDUFV3**	-	-	-	3	25%	96
**ND4**	4	7%	149/33	-	-	-
**ND5**	3	7%	84/32	-	-	-
**ACADVL**	12	25%	796/33	16	30%	777
**(NDUFAF2)**	4	22%	80/33			

Table S2. Peptide-based protein identification of the composition of *wild-type* complex I purified from heart. The number of peptides detected from each subunit is given together with the sequence coverage, relative to the sequence of the immature protein. The score given for each subunit is the sum of the peptide scores, with the denominator representing the 95% confidence threshold ($p < 0.05$). For proteins labelled in brackets individual peptide scores were below the 95% threshold but their protein scores were above the peptide threshold. Subunits NDUFC1, ND1, ND2, ND3, ND4L and ND6 (as well as ACAD9) were not detected.

	Ndufs4 $^{-/-}$ kidney	*Ndufs4* $^{-/-}$ heart				
		**Class 1**	**Class 1 ACADVL**	**Class 2**	**Class 2 N-module**	**Class 3**
PDB code	-	8C2S	8CA1	8CA3	8CA4	8CA5
EMDB code	16514	16398	16515	16516	16517	16518
**Data collection and processing**						
Magnification (nominal)	59,000	64,000				
Voltage (kV)	300	300				
Electron exposure (e^{-}/Å^{2})	49.24	45				
Defocus range (μm)	-2.1 to -3.3	-1.5 to -2.9				
Calibrated Pixel size (Å)	1.39	1.352				
Symmetry imposed	C1	C1	C2	C1	C1	C1
Initial particle images	149,198	1,141,407				
Final particle images	7,563	8,793	8,793	50,914	50,914	10,005
Map sharpening B-factor (Å^{2})	-130	-10	-27	-27	-10	0
Map resolution (FSC = 0.143) (Å)	6.2	3.9	4.3	3.2	3.2	3.9
**Model Refinement**						
Initial model used		6ZR2	AF-P50544	6ZR2	6ZR2	6ZR2<br>AF-Q59J78
Model resolution (FSC = 0.5) (Å)		3.7	4.2	3.1	3.2	3.7
Model composition<br>Non-hydrogen atoms<br>Protein residues<br>Ligands		 63,713<br>7,799<br>32	 9,036<br>1,178<br>2	 63,713<br>7,799<br>32	 11,113<br>1,439<br>6	 64,198<br>7,851<br>31
B factors mean (Å^{2})<br>Protein<br>Ligand		 62.57<br>63.68	 102.07<br>98.64	 51.34<br>60.08	 56.77<br>5.39	 76.25<br>77.85
RMSD Bond lengths (Å)<br>RMSD Bond angles (°)		0.007<br>1.071	0.006<br>1.185	0.005<br>0.995	0.005<br>1.017	0.004<br>0.658
Validation<br>MolProbity score<br>Clashscore<br>Poor rotamers (%)		 1.94<br>10.15<br>0.16	 1.89<br>9.75<br>0	 1.68<br>6.14<br>0.06	 1.87<br>7.44<br>0.25	 1.90<br>10.21<br>0.25
EMRinger score		2.15	2.19	3.03	3.01	2.19
Ramachandran plot<br>Favored (%)<br>Allowed (%)<br>Disallowed (%)<br>Z-score (whole)		 93.80<br>6.07<br>0.13<br>0.35	 94.38<br>5.45<br>0.17<br>1.92	 95.12<br>4.85<br>0.04<br>0.98	 92.65<br>7.28<br>0.07<br>0.34	 94.54<br>5.34<br>0.12<br>0.84
Map-model correlation CC_{mask}		0.82	0.82	0.87	0.89	0.78

Table S3. Cryo-EM data collection, refinement and validation statistics for maps and models of *ndufs4*$^{-/-}$ complex I and associated proteins.

	***Wild-type* active** EMD-11377	***Wild-type* deactive** EMD-11810
***ndufs4$^{-/-}$* class 1** EMD-16398	94	81-83
***ndufs4$^{-/-}$* class 2** EMD-16516	95-97	81-85
***ndufs4$^{-/-}$* class 3** EMD-16518	94-95	87-88

Table S4. Map-map correlations for consensus *ndufs4$^{-/-}$* class 1-3 maps against reference *wild-type* active and deactive maps. All maps were first lowpass-filtered to 3.9 Å. Maps thresholds were class 1, 2.07; class 2, 1.83; class 3, 1.85; *wild-type* active, 0.022; *wild-type* deactive, 0.04.
