# Supplementary material for: Structural insights into respiratory complex I deficiency and assembly from the mitochondrial disease-related ndufs4−/− mouse
Source: EMBO J. 2024 Jan 2;43(2):4. doi: 10.1038/s44318-023-00001-4 (PMC10897435; doi:10.1038/s44318-023-00001-4)
Supplement: Supplementary file 2 — Movie EV1 [file 44318_2023_1_MOESM2_ESM.zip › Movie EV1 legend.docx]

Movie EV1.

Relative movement between the *wild-type* and *ndufs4^-/-^* heart class 2 models, and motion within the cryo-EM density of *ndufs4^-/-^* heart class 2 from 3DVA analyses.
