# Supplementary material for: Structural insights into respiratory complex I deficiency and assembly from the mitochondrial disease-related ndufs4−/− mouse
Source: EMBO J. 2024 Jan 2;43(2):4. doi: 10.1038/s44318-023-00001-4 (PMC10897435; doi:10.1038/s44318-023-00001-4)
Supplement: Supplementary file 8 — Expanded View Figures [file 44318_2023_1_MOESM8_ESM.pdf]

Expanded View Figures

Figure EV1. Complexome profiles from BN-PAGE analyses of solubilized mitochondrial membranes from (A) wild-type and (B) *ndufs4*$^{-/-}$ mouse heart.

The heatmap represents the relative protein abundance in each gel slice (based on the peak areas of the three top-scoring peptides for each protein from LC-MS analyses) with the data for each protein normalized to the highest intensity signal within the lane. The prefix "NDU" has been omitted from subunit labels for brevity. Where a protein was not detected in one sample, the column is completely black. Subunits ND3, ND4L and ND6 were not detected in either sample. The masses of complex I-related bands specific to *ndufs4*$^{-/-}$ membranes of ~800 (QP subcomplex) and ~200 kDa (N module) were estimated using the migration and masses of known complexes (inset). Source data are available online for this figure.

 © The Author(s)

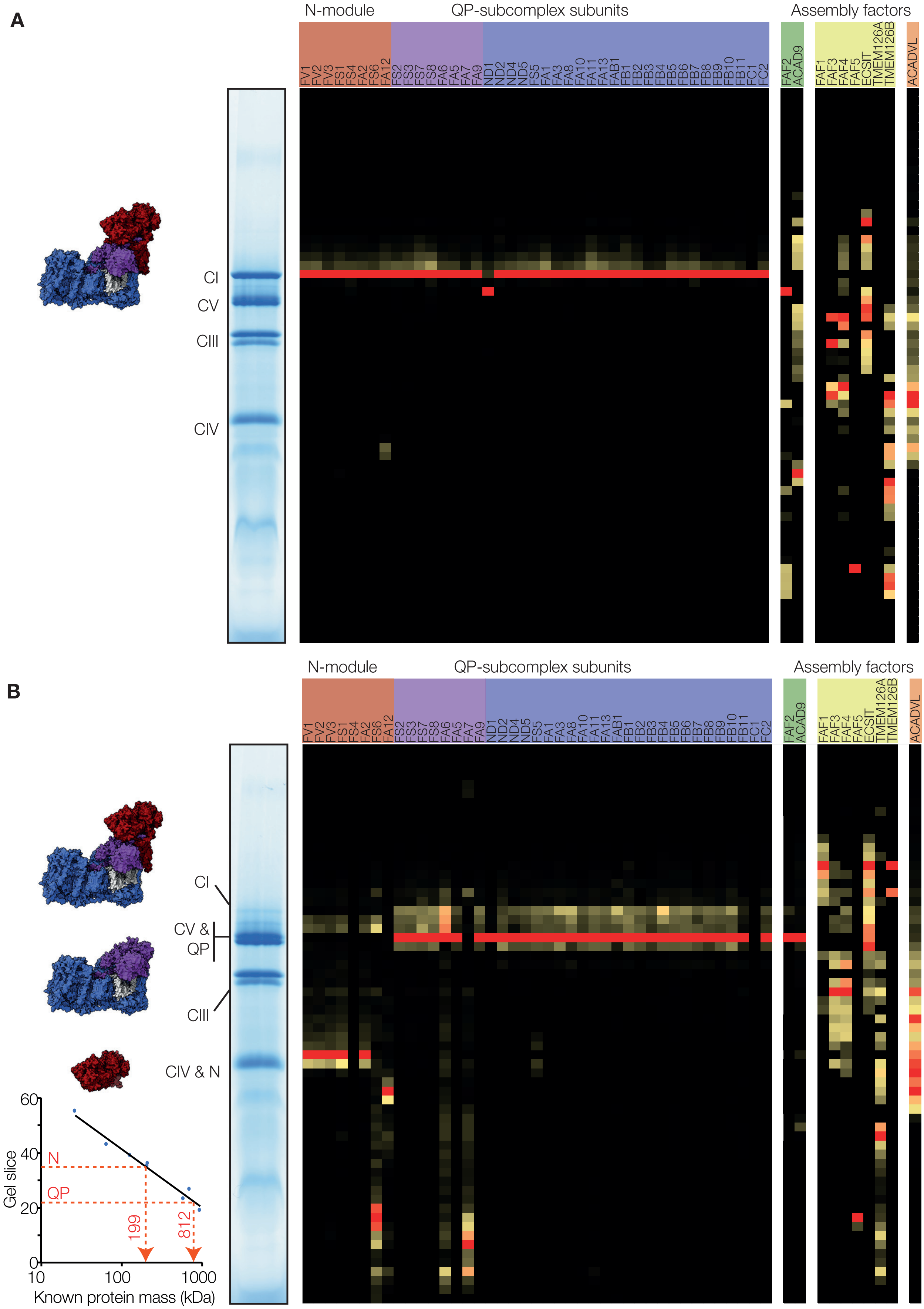
A
N-module
QP-subcomplex subunits
Assembly factors
FV1 FV2 FV3 FS1 FS4 FA2 FS6 FA12 FS2 FS3 FS7 FS8 FA6 FA5 FA7 FA9 ND1 ND2 ND4 ND5 FS5 FA1 FA3 FA8 FA10 FA11 FA13 FAB1 FB1 FB2 FB3 FB4 FB5 FB6 FB7 FB8 FB9 FB10 FB11 FC1 FC2
FAF2 ACAD9
FAF1 FAF3 FAF4 FAF5 ECSIT TMEM126A TMEM126B
ACADVL
CI
CV
CIII
CIV
B
N-module
QP-subcomplex subunits
Assembly factors
CI
CV & QP
CIII
CIV & N
Gel slice
Known protein mass (kDa)
N
QP
199
812

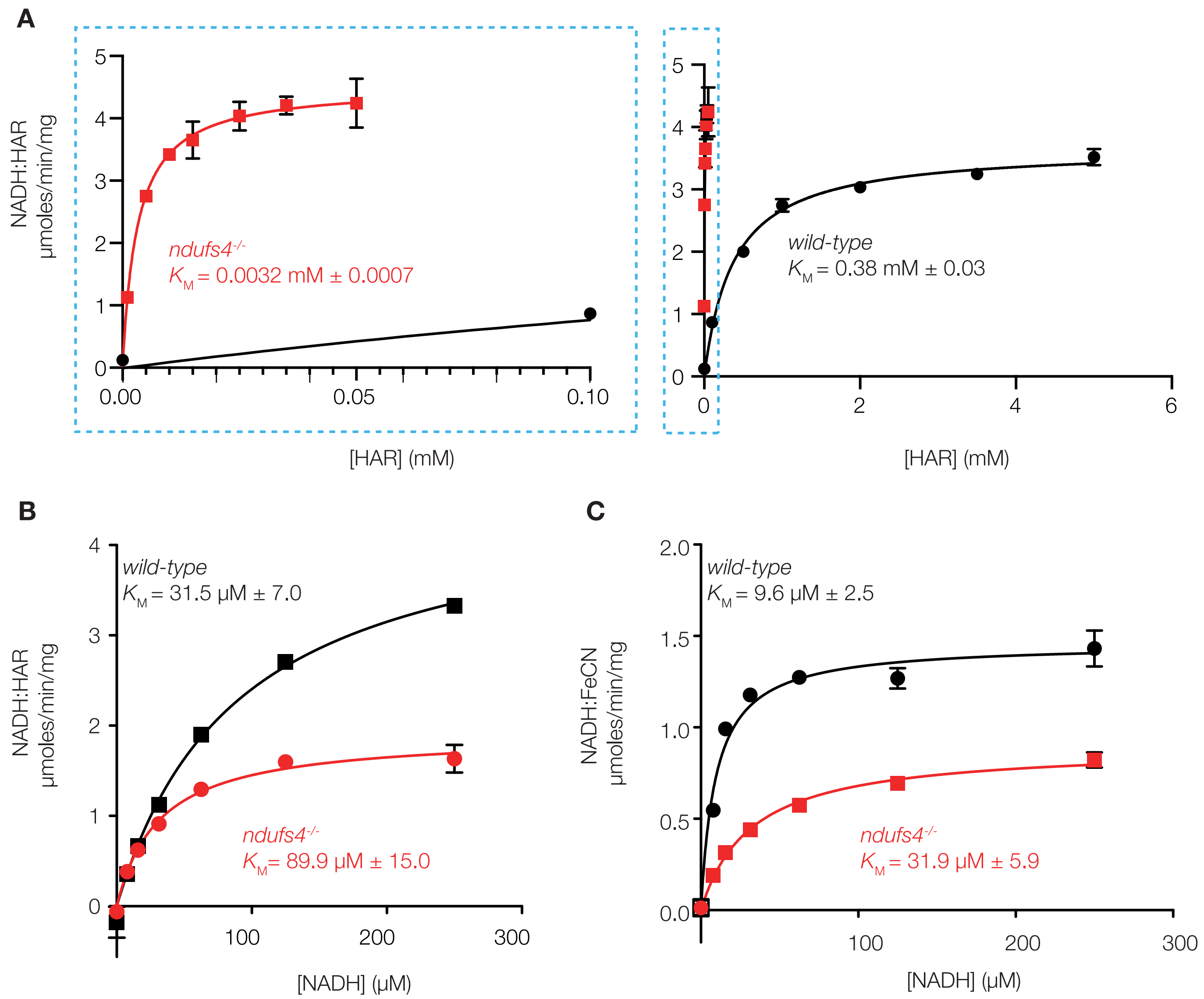

Figure EV2. Flavin-site kinetics in *ndufs4*$^{-/-}$ mitochondrial membranes compared to wild-type.

(**A**) Kinetic evaluation of the Michaelis constant (K_M) for HAR in the NADH:HAR oxidoreductase reaction catalyzed by mouse heart membranes in the presence of 100 µM NADH. (**B**) Kinetic evaluation of the Michaelis constant (K_M) for NADH in the presence of 1 mM HAR. (**C**) Kinetic evaluation of the Michaelis constant (K_M) for NADH in the NADH:FeCN oxidoreductase reaction catalyzed by mouse kidney membranes in the presence of 100 µM FeCN. Due to the scarcity of materials, the assays in panels **A** and **B** were performed on different membrane samples, so the absolute rates are not directly comparable between them. Data points are mean averages ± SEM (n = 3 technical replicates). Source data are available online for this figure.

 © The Author(s)

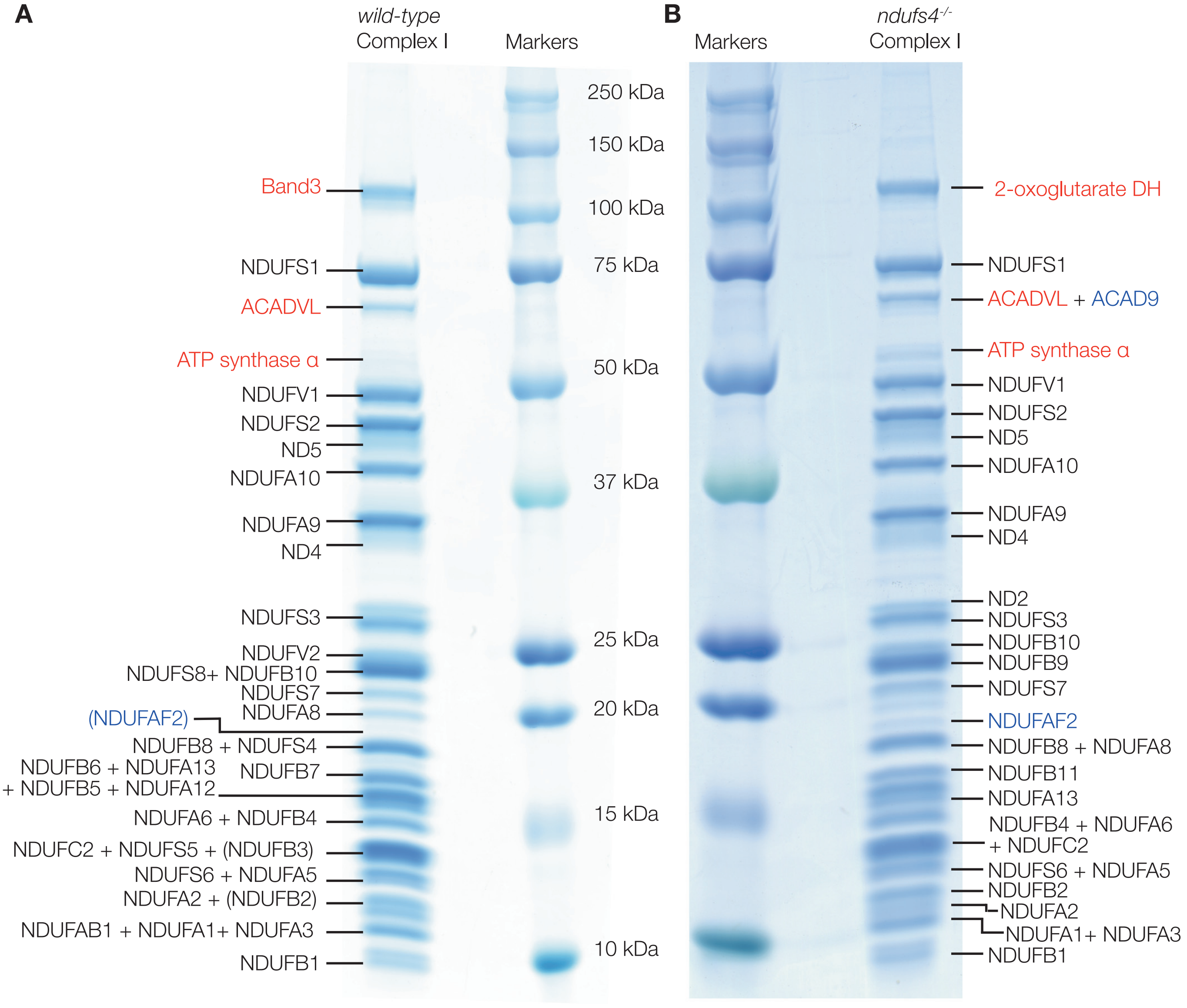

Figure EV3. SDS PAGE of complex I purified from (A) wild-type and (B) *ndufs4*$^{-/-}$ heart mitochondrial membranes.

Bands were manually excised, and proteins identified by MALDI-TOF-TOF analyses of tryptic digests. Complex I subunits are labeled in black, known assembly factors in blue, and other proteins detected in red. For proteins labeled in brackets individual peptide scores were below the 95% threshold but their protein scores were above the peptide threshold. Information on protein identification is in Appendix Tables S1 and S2.

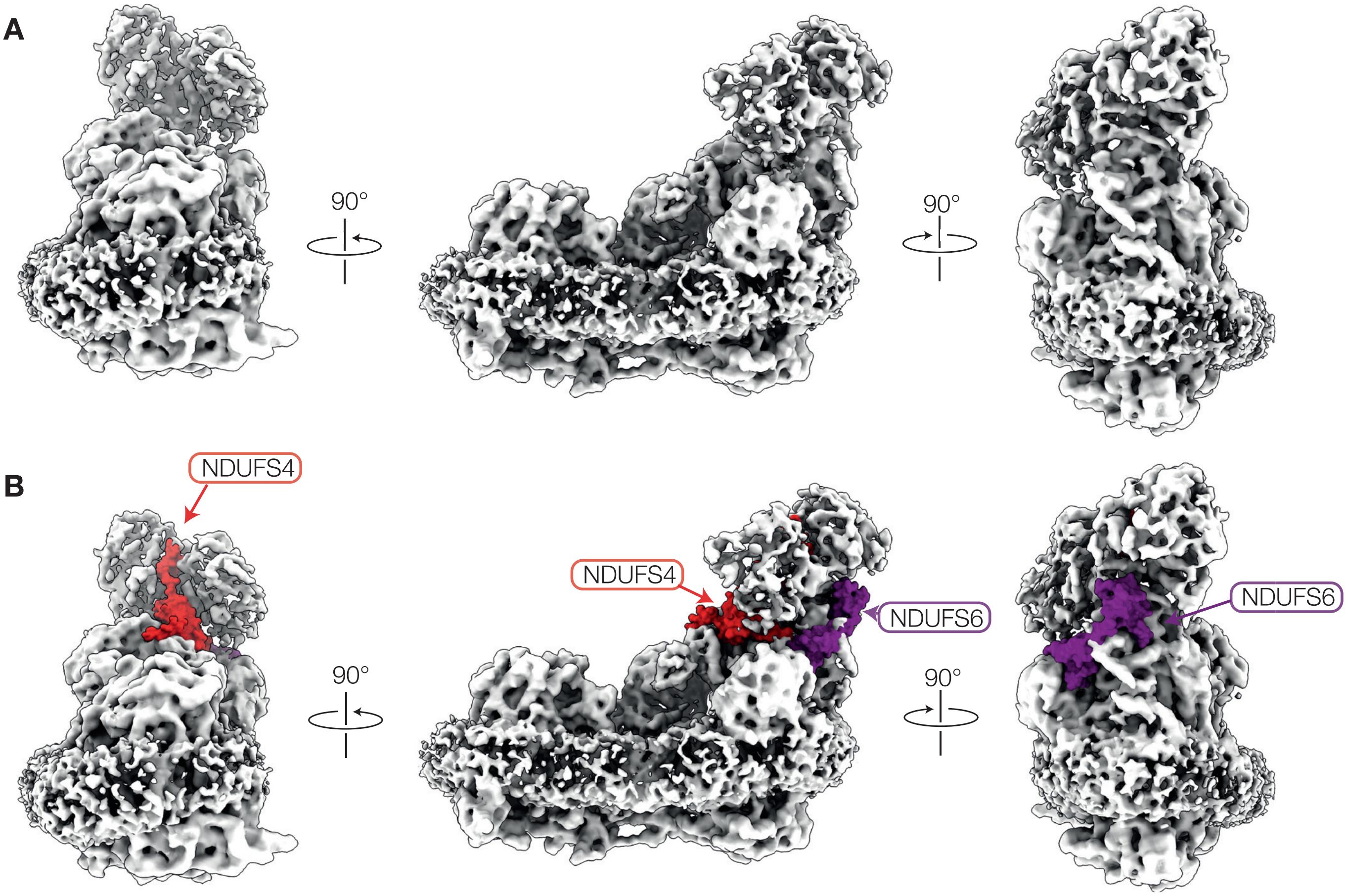

Figure EV4. Views of the cryo-EM density map of complex I from *ndufs4*$^{-/-}$ kidney and comparison to the model for wild-type heart complex I.

(**A**) The density map of complex I from *ndufs4*$^{-/-}$ kidney. (**B**) The wild-type complex I model (PDB:6ZR2) is docked into the *ndufs4*$^{-/-}$ complex I density map (gray, EMD:16514); no density is present in the locations of NDUFS4 (red model) and NDUFS6 (purple model).

 © The Author(s)

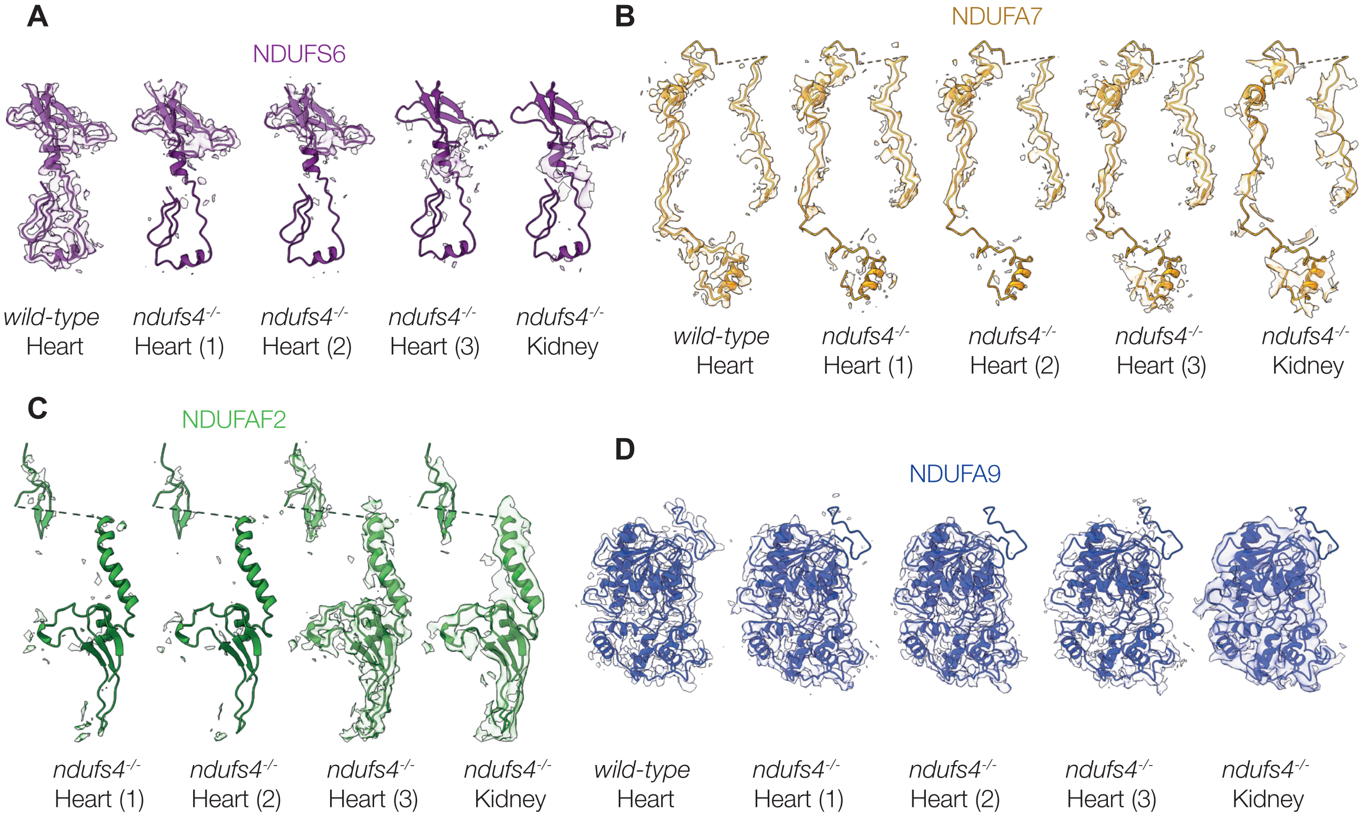

Figure EV5. The cryo-EM densities for NDUFS6, NDUFA7, NDUFAF2 and NDUFA9.

The models for wild-type heart complex I (PDB: 6ZR2, for NDUFS6, NDUFA7 and NDUFA9) and the class 3 model (for NDUFAF2) from were fitted into each density map using the fit-in-map function in Chimera X. The density within 3 Å of each fitted model is shown for (**A**) subunit NDUFS6; (**B**) subunit NDUFA7; (**C**) assembly factor NDUFAF2; and (**D**) subunit NDUFA9. They are shown for the map of wild-type heart complex I (EMD-11377); the *ndufs4*$^{-/-}$ heart complex I class 1, 2 and 3 maps; and the *ndufs4*$^{-/-}$ kidney complex I map, with map thresholds of 0.027, 1.76, 2.18, 1.52 and 1.12, respectively.

© The Author(s)
